# Synthetic Covalently Linked Dimeric Form of H2 Relaxin Retains Native RXFP1 Activity and Has Improved *In Vitro* Serum Stability

**DOI:** 10.1155/2015/731852

**Published:** 2015-01-22

**Authors:** Vinojini B. Nair, Ross A. D. Bathgate, Frances Separovic, Chrishan S. Samuel, Mohammed Akhter Hossain, John D. Wade

**Affiliations:** ^1^Florey Institute of Neuroscience and Mental Health, University of Melbourne, Parkville, VIC 3010, Australia; ^2^School of Chemistry, University of Melbourne, Parkville, VIC 3010, Australia; ^3^Department of Biochemistry and Molecular Biology, University of Melbourne, Parkville, VIC 3010, Australia; ^4^Department of Pharmacology, Monash University, Clayton, VIC 3800, Australia

## Abstract

Human (H2) relaxin is a two-chain peptide member of the insulin superfamily and possesses potent pleiotropic roles including regulation of connective tissue remodeling and systemic and renal vasodilation. These effects are mediated through interaction with its cognate G-protein-coupled receptor, RXFP1. H2 relaxin recently passed Phase III clinical trials for the treatment of congestive heart failure. However, its *in vivo* half-life is short due to its susceptibility to proteolytic degradation and renal clearance. To increase its residence time, a covalent dimer of H2 relaxin was designed and assembled through solid phase synthesis of the two chains, including a judiciously monoalkyne sited B-chain, followed by their combination through regioselective disulfide bond formation. Use of a bisazido PEG_7_ linker and “click” chemistry afforded a dimeric H2 relaxin with its active site structurally unhindered. The resulting peptide possessed a similar secondary structure to the native monomeric H2 relaxin and bound to and activated RXFP1 equally well. It had fewer propensities to activate RXFP2, the receptor for the related insulin-like peptide 3. In human serum, the dimer had a modestly increased half-life compared to the monomeric H2 relaxin suggesting that additional oligomerization may be a viable strategy for producing longer acting variants of H2 relaxin.

## 1. Introduction

Relaxin, one of the first hormones to be discovered, is a member of the insulin superfamily of peptides [1]. It is a small two-chain, three-disulfide bonded peptide [2, 3]. Once much ignored by the international research community [4], relaxin, similar to its sister hormones insulin and the insulin-like growth factors (IGFs), is now known as a multifunctional hormone. It is primarily involved with the maintenance of reproduction and pregnancy and in the facilitation of the delivery of the young. Its native G-protein-coupled receptor, relaxin family peptide receptor 1 [5], RXFP1 (previously known as LGR7), was shown to be widely distributed in various organs in both males and females. Human (H2) relaxin, the major stored and circulating form of human relaxin, is now known to play a key role in inflammatory and matrix remodeling processes and possesses potent vasodilatory, angiogenic, and other cardioprotective actions [6, 7].

At physiological concentrations, the H2 relaxin exists as a monomer [3]. However, Eigenbrot et al. reported the crystal structure for H2 relaxin and showed that it exists as a noncovalent dimer [8]. This was confirmed by sedimentation equilibrium analytical ultra-centrifugation studies [9]. This probably corresponds to the stored form of H2 relaxin and such a dimer is likely to be biologically inactive because the known key receptor binding residues (R^B13^, R^B17^, and I^B20^) in H2 relaxin [2, 7] form part of the dimer interface [8]. This is supported by the fact that the monomer of the related peptide, insulin, is involved in its tyrosine kinase receptor activation whereas its dimeric and hexameric forms are involved in stabilizing the molecule during storage [10, 11]. A covalently linked (through a disulfide bridge) dimeric insulin peptide was recently prepared by recombinant DNA technology. It neither bound to the insulin receptor nor induced a metabolic response* in vitro* [12], which is consistent with the view that the monomeric form is the active form of insulin. However, the insulin dimer was shown to be extremely thermodynamically stable* in vitro* which highlighted the importance of oligomerization for insulin stability [12]. This suggests that a covalently linked dimeric relaxin may also be more stable* in vitro* as well as* in vivo* compared to its monomeric form. Such a compound, if it retained biological activity, would be very valuable given that H2 relaxin recently passed Phase III clinical trials for treating acute heart failure [13] despite having a short* in vivo* half-life of approximately 10 min [14, 15] that is characteristic of many peptides and proteins. It is for this reason that H2 relaxin requires continuous intravenous infusion into patients over 48 hours [13]. Therefore, there is a clear need for improving the pharmacokinetic properties of H2 relaxin in order to potentially improve its therapeutic value.

**Figure 1:**
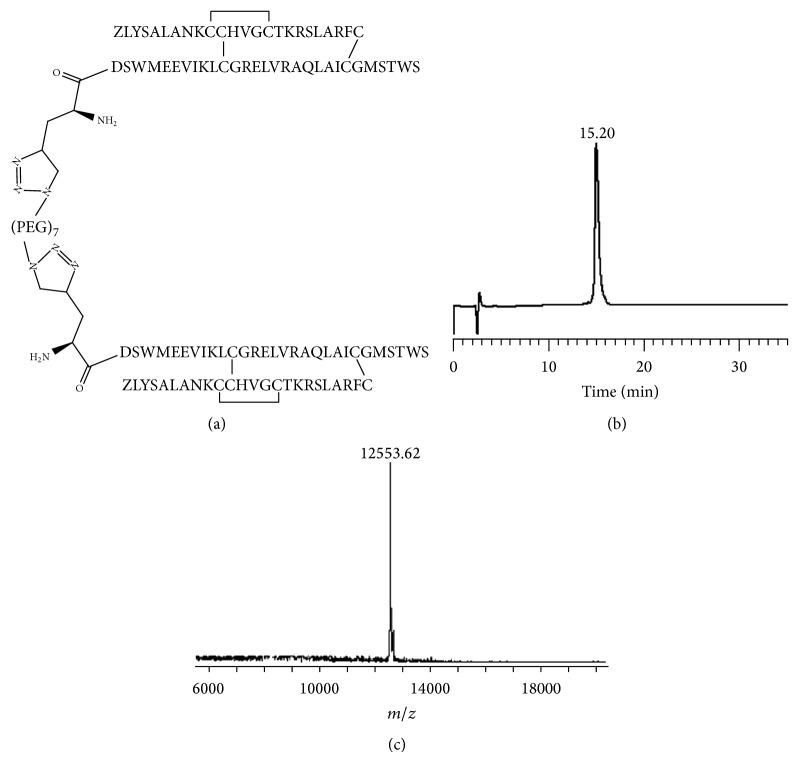
(a) Primary structure and (b) RP-HPLC and (c) MALDI-TOF MS traces of synthetic H2 (PEG)_7_ H2 dimer. Theoretical [M + Na]^+^: 12,530.0.

In this study, we undertook to design and develop a covalently linked dimeric analogue of synthetic H2 relaxin using both click chemistry and a small polyethylene glycol (PEG) spacer to link the two monomers in such a structural orientation so as to retain biological activity (Figure 1). We studied its structural and functional role using circular dichroism (CD) spectroscopy and RXFP1- and RXFP2-expressing cells, respectively. It was shown that the dimeric H2 relaxin possessed a high degree of secondary structural similarity to native H2 relaxin. Importantly, unlike the insulin dimer, the dimeric H2 relaxin is equipotent to native H2 relaxin monomer and exhibits improved* in vitro* serum stability.

## 2. Experimental Procedures

### 2.1. Materials

9-Fluorenylmethoxycarbonyl- (Fmoc-) protected L-*α*-amino acids and 1-[bis(dimethylamino)methylene]-1*H*-benzotriazolium hexafluorophosphate 3-oxide (HBTU) were purchased from GL Biochem (Shanghai, China). N,N-Dimethylformamide (DMF), piperidine, and trifluoroacetic acid (TFA) were obtained from Auspep (Melbourne, Australia). PAL-PEG-PS resins with substitution of ca. 0.20 mmol/g were purchased from Applied Biosystems Inc. (Melbourne, Australia). Diethyl ether, methanol, acetonitrile, and dichloromethane were purchased from Merck (Melbourne, Australia), trifluoromethanesulfonic acid (TFMSA), anisole, triisopropylsilane (TIPS), 3,6-dioxa-1,8-octanedithiol (DODT), and diisopropylethylamine (DIEA) were from Sigma-Aldrich (Sydney, Australia), and 2,2′-dipyridyl disulfide (DPDS) was from Fluka (Switzerland). CaCl_2_ was purchased from Mallinckrodt (Melbourne, Australia) and Na_2_HPO_4_ and MgSO_4_ were purchased from BDH Chemicals while KH_2_PO_4_ and MnCl_2_ were purchased from Ajax Chemicals (Sydney, Australia). Dulbecco's modified Eagle medium (DMEM), penicillin/streptomycin, L-glutamine, and fetal bovine serum were purchased from Invitrogen (Melbourne, Australia). Sterile-filtered serum from human AB plasma was purchased from Sigma-Aldrich (Sydney, Australia). All other reagents were purchased from Sigma-Aldrich (Sydney, Australia). Native recombinant DNA-derived human relaxin-2 used as control was obtained from Corthera Inc. (San Francisco, CA, a subsidiary of Novartis AG, Basel, Switzerland). Synthetic europium-labelled H2 relaxin, unlabelled H2, and human INSL3 were prepared in-house as previously described [16, 17].

### 2.2. Synthesis

#### 2.2.1. Chemical Peptide Synthesis

The H2 relaxin A- and B-chains were assembled as C-terminal amides on an automated Protein Technologies Tribute peptide synthesizer (Tuscan, AZ) or CEM Liberty microwave peptide synthesizer (Ai Scientific, Australia) using Fmoc chemistry. Side chain protecting groups of trifunctional amino acids were TFA-labile, except for* tert*-butyl- (tBu-) protected cysteine in position A11 and acetamidomethyl- (Acm-) protected cysteines in positions A24 and B23. Using instrument default protocols, both A- and B-chains were separately synthesized either at 0.1 mmol scale or 0.2 mmol scale activated with either 4- or 5-fold molar excess of HBTU (0.4 or 0.5 mmol for a 0.1 mmol scale of resin; 0.8 mmol for a 0.2 mmol scale of resin) in the presence of 5 equivalents DIEA. Resin-attached peptides were treated with 20% v/v piperidine/DMF to remove N^*α*^-Fmoc protecting groups. When using the microwave synthesizer, coupling and deprotection steps were carried out at 75°C and 25 W microwave power for 5 mins and 60 W microwave power for 3 mins, respectively, when using the CEM Liberty microwave synthesizer. Coupling and deprotection steps were carried out for 30 and 10 mins, respectively, when using Tribute synthesizers. Upon complete coupling of the final amino acid of the native B-chain peptide sequence, an extra amino acid containing the alkyne group (Fmoc-L-propargylglycine) was coupled by manual coupling procedures.

#### 2.2.2. Peptide-Resin Cleavage

Upon completion of solid phase synthesis, the A- and B-chains were cleaved from the solid supports by treatment with TFA containing anisole/TIPS/DODT (94%/2.5%/2%/1.5%, 20 mL) for 2 h. Cleaved products were concentrated by N_2_ bubbling, precipitated with ice-cold diethyl ether and centrifuged at 3000 rpm for 5 mins. The centrifuged pellet was then washed with ice-cold diethyl ether and centrifuged. This process was repeated at least three times.

#### 2.2.3. Peptide Purification

All RP-HPLC analytical and purification reactions were monitored using analytical and preparative Vydac C18 columns (pore size, 300 Å; particle size 4.6 × 250 mm or 22 × 250 mm, resp.), in a gradient mode with eluant A: 0.1% aq TFA and eluant B: 0.1% TFA in acetonitrile.

#### 2.2.4. Preparation of Monoalkyne H2 Relaxin

Following simultaneous cleavage and side chain deprotection and purification of the crude A- and B-chains, stepwise formation of the three disulfide bonds was carried out via successive oxidation, thiolysis, and iodolysis as previously described [18].

#### 2.2.5. Formation of H2 (PEG)_7_ H2 Dimer

PEG_7_ bisazide (Jena Bioscience, Germany) was coupled onto alkyne H2 relaxin employing copper-catalyzed click reaction. The alkyne peptide was first dissolved in phosphate buffer saline (PBS) at pH 7.0. Following this, 0.5 equivalents of PEG_7_ bisazide and 20 equivalents of aqueous copper (II) sulfate pentahydrate were added sequentially. Finally, 21 equivalents of aqueous ascorbic acid (made up fresh for use) were added and reaction left stirring at room temperature. This reaction progress was monitored by analytical RP-HPLC and stopped after 45 minutes by diluting with approximately 100 *μ*L deionised water before RP-HPLC purification.

#### 2.2.6. Peptide Characterization by MALDI-TOF MS

Synthetic peptides (from intermediate steps and final product) were confirmed by matrix-assisted laser desorption ionization time-of-flight mass spectrometry (MALDI-TOF MS) utilizing a Bruker Ultraflex II instrument (Bruker Daltonics, Bremen, Germany) using sinapinic acid (3,5-dimethoxy-4-hydroxycinnamic acid) matrix. This matrix was made up in 70% acetonitrile containing 0.1% TFA.

#### 2.2.7. Peptide Quantitation by Amino Acid Analysis

Peptide content and amino acid composition were determined using vapour phase acid hydrolysis in 6 M HCl/2% phenol at 110°C for 24 h. Individual amino acids were converted to stable, fluorescent derivatives using Waters AccQTag kit (Waters, Sydney, Australia). Derivatized amino acids were separated using Shim-Pak XR-ODS (3 × 75 mm, 2.2 *μ*m) on Shimadzu RP-HPLC system (Shimadzu, Victoria, Australia).

#### 2.2.8. Secondary Structure Analysis by CD Spectroscopy

A JASCO instrument (J-185, Tokyo, Japan) was utilized to obtain CD spectral analysis of all peptides. The following settings were used to obtain readings: wavelength range 195 to 250 nm, scanning speed 50 nm/min, bandwidth 0.1 nm, and cell length 1 mm at 25°C. The peptide samples were prepared at 0.2 *μ*g/*μ*L in 10 mM PBS, pH 7.4. Raw data from the spectra in millidegree of ellipticity (*θ*) were converted to mean residual weight ellipticity (MRE) [19].

#### 2.2.9. Receptor Binding Assays

HEK-293T cells stably transfected with RXFP1 grown in DMEM medium supplemented with 10% FBS, 100 *μ*g/mL penicillin, 100 *μ*g/mL streptomycin, and 2 mM L-glutamine were plated out at 40 000 cells per well per 200 *μ*L in a 96-well ViewPlate with clear bottom and white walls precoated with poly-L-lysine. Competition binding experiments were performed with 5 nM europium-labelled H2 relaxin [17] in the absence or presence of increasing concentrations of unlabelled peptides. Nonspecific binding was determined with an excess of unlabelled peptides (500 nM H2 relaxin). Fluorescent measurements were carried out at excitation of 340 nm and emission of 614 nm on a Victor Plate reader (Perkin-Elmer, Melbourne, Australia). All data are presented as the mean ± S.E. of the total specific binding percentage (in triplicate wells), repeated in at least three independent experiments and curves fitted using one-site binding curves in GraphPad Prism 5 (GraphPad Inc., San Diego, CA). Statistical differences in pIC50 values were analyzed using one-way analysis of variance (ANOVA) coupled to a Newman-Keuls multiple comparison test for multiple group comparisons in GraphPad Prism 5.

#### 2.2.10. Functional cAMP Assay

The influence of cAMP signaling by synthetic analogues in HEK-293T cells expressing either human RXFP1 or RXFP2 receptors was assessed using cAMP reporter gene assay as described previously [20]. Briefly, HEK-293T cotransfected with response element pCRE *β*-galactosidase reporter plasmid were plated out in Corning Cell BIND 96-well plate at 50 000 cells per well per 200 *μ*L. 24 h later, cotransfected cells were treated with increasing concentrations of H2 relaxin analogues in parallel with native H2 relaxin or human INSL3. After 6 h incubation at 37°C, cell media were aspirated and cells were frozen at −80°C overnight. A *β*-galactosidase colorimetric assay measuring absorbance at 570 nm on Benchmark Plus microplate spectrophotometer (Bio-Rad, Gladesville, Australia) was then used to measure relative cAMP responses. Each concentration point was measured in triplicate and each experiment performed independently at least three times. Ligand-induced stimulation of cAMP was expressed as a percentage of the maximum H2 relaxin response for RXFP1 cells or human INSL3 for RXFP2 cells. GraphPad Prism 5 was used to analyze the cAMP activity assay which was expressed as the mean ± S.E.M. Statistical analysis was conducted using one-way ANOVA with Newman-Keuls post hoc analysis.

#### 2.2.11. *In Vitro* Serum Stability

The stability of the synthetic analogue was measured against native H2 relaxin in human serum. The purchased serum was not heat inactivated in order to retain as much of its proteolytic enzymic activity as possible. The peptides tested were normalized to a final concentration of 1.0 mg/mL with deionized water, and 10.0 *μ*L of peptides was added to 590 *μ*L 100% human serum. After addition of peptide into the serum, 50 *μ*L of samples was removed and quenched with 250 *μ*L of ice-cold acetonitrile/0.1% TFA. The solution was then spun down with a benchtop centrifuge at 13,500 rpm (Eppendorf Centrifuge 5804R, Melbourne, Australia) for 15 mins at 4°C to pellet the larger, precipitated serum proteins. The remaining peptide/serum solution was placed immediately into 37°C incubator. Degradation of peptides was monitored with manual RP-HPLC injections of supernatant using a Phenomenex Aeris Widepore 3.6 *μ*m C4 analytical column (pore size, 100 Å; particle size 4.6 × 250 mm), in a stabilized gradient mode with eluent A: 0.1% aq TFA and eluant B: 0.1% TFA in acetonitrile. Samples were taken at time points 0.5, 1.0, 1.5, 2.0, 2.5, 3, 4, 5, and 6 h. Each assay was carried out in triplicate. Elution of target peptide was elucidated by retention time analysis and characterization with MALDI-TOF MS at each time point. Kinetic analysis of target peptide at each time point was carried out by least squares analysis of the logarithm of the integration peak area versus retention time. Nonspecific peptide degradation was measured by peptide degradation in serum at 0 h. Correction for serum peptides that might coelute with target peptides was carried out by subtracting integrated peak areas with equivalent serum only solution at all time points (background subtraction). GraphPad Prism 5 was used to analyze the peptide degradation in serum which was expressed as the mean ratio ± S.E.M. and Microsoft Excel was used to graph all data. Statistical analysis was conducted using one-way ANOVA with Newman-Keuls post hoc analysis.

## 3. Results

**Figure 2:**
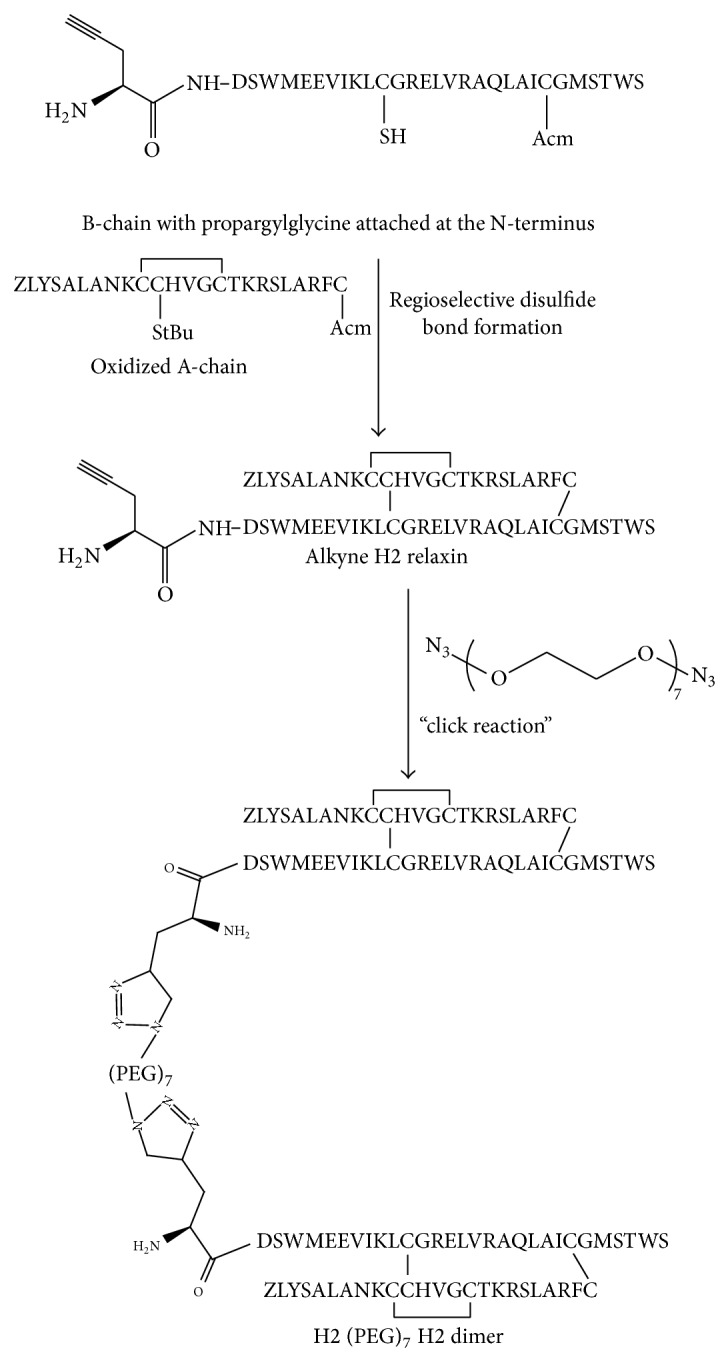
Scheme for the preparation of H2 (PEG)_7_ H2 dimer by solid phase peptide synthesis and click chemistry.

The selectively S-protected A- and B-chains were of high purity as determined by analytical RP-HPLC and MALDI-TOF MS. An Fmoc-propargylglycine was manually coupled onto the N-terminal end of the B-chain prior to cleavage from the solid support and subsequent crude peptide purification. Following this, stepwise regioselective disulfide bond formation between the A-chain and monoalkyne molecule-chain was carried out according to established protocols [21–23] to form the two-chain, three-disulfide bond alkyne H2 relaxin. Copper-catalyzed alkyne-azide cycloaddition was then utilized to “click” two molecules of alkyne-H2 relaxin onto a bisazido PEG, (PEG)_7_, hence forming a dimeric relaxin molecule separated by a short PEG spacer (Figure 2). This was termed H2 (PEG)_7_ H2 dimer and was shown to be of high purity as assessed by analytical RP-HPLC and MALDI-TOF MS (Figure 1).

**Figure 3:**
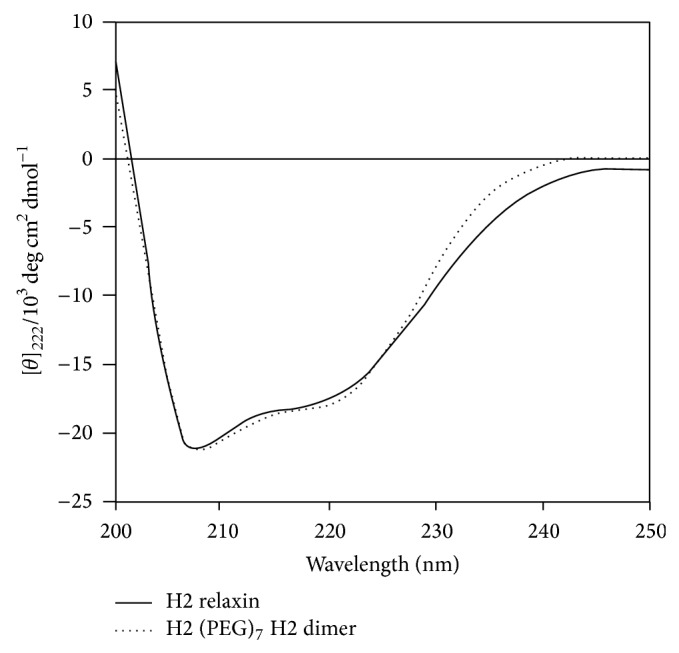
CD spectroscopic data of the H2 (PEG)_7_ H2 dimer in comparison with native H2 relaxin.

The CD spectra of H2 (PEG)_7_ H2 were measured in 10 mM PBS (pH 7.4) buffer (Figure 3). The dimer was found to retain a very similar secondary structure and a high degree of *α*-helical conformation (with pronounced double minima at approximately 208 nm and 222 nm) along with some *β*-sheet and random coiled structure. The *α*-helical content of H2 relaxin was found to be 49% helicity compared to the H2-PEG dimer with 48%. These values were calculated from the MRE at 222 nm, the [*θ*]_222_ values for relaxin and H2 (PEG)_7_ H2 dimer being −17511.4 and −17679.4, respectively. The similarities between the MRE and helix content between these two peptides suggested that the dimer essentially retained native H2 relaxin-like structure.

**Figure 4:**
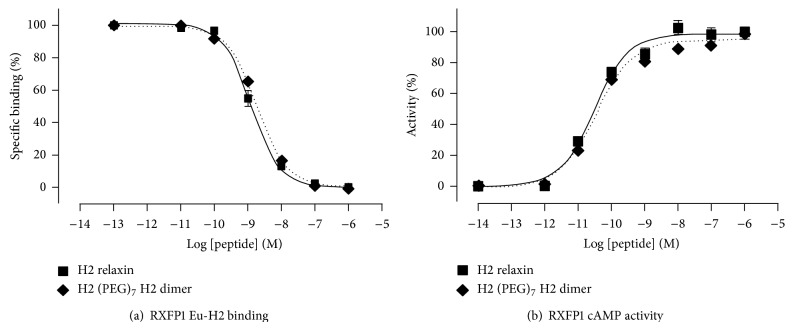
Binding and activity of synthetic H2 (PEG)_7_ H2 dimer in comparison to native H2. (a) Competition binding of H2 (PEG)_7_ H2 dimer with europium-labeled H2 relaxin in HEK-293T cells stably expressing RXFP1. (b) cAMP activity in RXFP1 expressing HEK-293T cells using a pCRE-*β*-galactosidase reporter gene system. Data are expressed as percentage of cAMP response and are pooled data from at least three independent experiments performed in triplicate.

**Figure 5:**
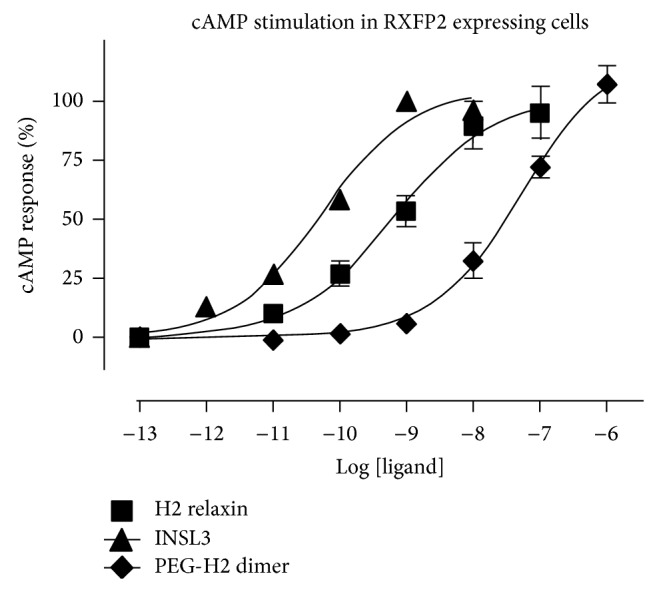
cAMP activity in RXFP2 expressing HEK-293T cells using a pCRE-*β*-galactosidase reporter gene system. Data are expressed as percentage of cAMP response and are pooled data from at least three independent experiments performed in triplicate.

To assess the biological activity of the dimer, binding and activity* in vitro* assays were undertaken in comparison to the native recombinantly produced H2 relaxin. These assays were carried out in HEK-293T cells stably expressing RXFP1, the native receptor of H2 relaxin. The starting alkyne H2-relaxin was also assessed for binding and signaling through the RXFP1 and RXFP2 receptor. This was performed to confirm that the disulfide bonds within the two monomeric analogues were assembled in the correct form as other combinations of disulfide pairings have previously been shown to result in no interaction with the RXFP1 receptor (neither binding nor activity) [24]. Following confirmation of the alkyne monomer possessing full H2 relaxin-like activity (data not shown), the H2 (PEG)_7_ H2 dimer was tested for binding and cAMP activity on the same HEK-293T cells expressing RXFP1. It was found to have similar affinity and potency to H2 relaxin at the RXFP1 receptor (Figures 4(a) and 4(b)). The dimer was then also tested with RXFP2, the native receptor for INSL3. Interestingly, the peptide displayed significantly weaker activation propensity (Figure 5).

**Figure 6:**
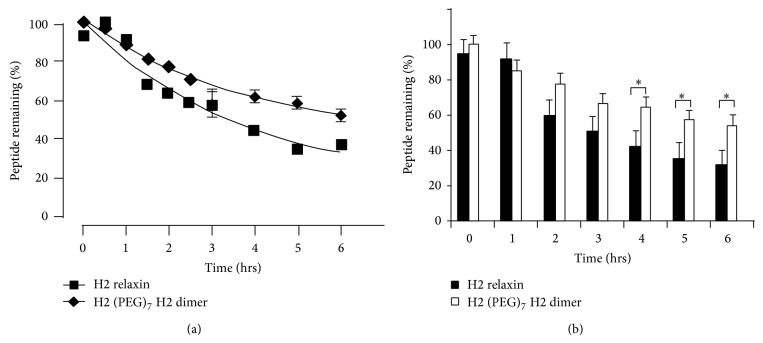
*In vitro* serum stability analysis. (a) Curve: half-life determination of H2 (PEG)_7_ H2 dimer when compared to H2 relaxin incubated* in vitro* at 37°C in human male serum. (b) Bar graph: comparison of peptide degradation at different time points. ^*^ *P* < 0.05.

The degradation kinetics and serum stability of the synthetic H2 (PEG)_7_ H2 dimer was measured against native H2 relaxin in male human serum (Figure 6). The purchased serum was not heat inactivated in order to retain as much activity of its proteolytic enzymes and other reductants as possible. This provided a better representation of human serum and a direct* in vitro* measurement in an experimental setting of the degradation kinetics of the relaxin dimer when compared to native relaxin. The H2 (PEG)_7_ H2 dimer was observed to retain its original dimeric form* in vitro* with a half-life of 2.52 hrs, significantly longer when compared to native relaxin with 2.22 hrs.

## 4. Discussion

Nearly 9 decades since its discovery, the pleotropic peptide H2 relaxin recently completed a successful Phase III clinical trial for the treatment of acute heart failure [13, 25]. Despite this important success, the short* in vivo* half-life of the peptide (10 mins) [14, 15] necessitates its continuous intravenous infusion for optimum activity. As the* in vivo* half-life of a peptide or protein is affected by both its degradation by enzymes and renal clearance [26, 27], it can be improved by, for example, conjugation with large MW compounds such as PEG or by polymerization/aggregation which acts to (at least partially) shield it from proteolytic enzymes and also by slowing clearance, although best results for the latter occur with a molecular mass of greater than 40 kDa [26]. Conjugation of PEG molecules to protein-based biopharmaceuticals has recently proved highly successful in increasing their therapeutic index and clinical use [28]. For example, a combinational treatment of PEGylated *α*-2-interferon and ribavirin has successfully eradicated hepatitis C virus in approximately 50% of the treated patients, leading to several PEG-based proteins being approved for therapeutic purposes [29]. PEG moieties are also highly hydrated, hence increasing the hydrodynamic radius of the conjugates and correspondingly improving solubility and reducing urinary glomerular filtration rate [30]. However, such modifications are not without disadvantages. The polydispersity of PEG can complicate accurate quality control as well as introducing physicochemical property modifications that make chemical characterization by RP-HPLC and MS extremely challenging due to peak broadening, even disappearance, and a lack of ionization. For this reason, oligomerization strategies are gaining increasing attention as a means of increasing* in vivo* stability [31]. A dimeric erythropoietin formed by chemical crosslinking of the monomer showed an increased plasma half-life in rabbits of more than 24 h compared to the monomer's 4 h. The dimer also possessed 26-fold higher activity* in vivo* [32]. A dimer of the antibacterial peptide, A3-APO, possessed a substantially increased serum half-life (100 min) compared to the monomer (4 min) [33]. For this reason, we undertook to examine the feasibility of developing a synthetic dimer of H2 relaxin as a first step towards obtaining improved pharmacokinetics. This was designed and assembled using a bisazido PEG_7_ moiety between two molecules of synthetic functionalized H2 relaxin via click chemistry. This linker was chosen because it is sufficiently longer to space the two peptide units apart and also because it is monodisperse thus simplifying characterization. Our previous studies have shown that the N-terminus of the B-chain can be truncated by six residues [34] or modified to accommodate a functional moiety such as a biotin [35] or large fluorophore [18] without significant loss of activity. This is because this site is far from its primary active site which consists of the B-chain C-terminal *α*-helical region [2]. Consequently, the bisazido PEG_7_ moiety was conjugated at both its ends with N-terminal B-chain alkyne H2 relaxin employing the now well-established click reaction.

The CD spectral data showed that H2 (PEG)_7_ H2 dimer had a comparable secondary structure to native H2 relaxin (Figure 3), strongly suggesting that the dimeric form of H2 relaxin has native relaxin-like structural integrity. Further evidence was also provided by the near-native RXFP1 receptor binding and activation activity (Figure 4). This result confirmed that the presentation of the active site of H2 relaxin is essentially unaffected by the tethering of the molecule to the PEG_7_ linker via the N-terminus of the B-chain. This is reflected by the* in vitro* RXFP1 binding and cAMP data in that despite the dimer being at least twice the molecular size of native relaxin, its size and spacer length did not impair the ability of the dimer to bind and activate downstream RXFP1 signaling. The RXFP1 binding domain within the B-chain of the dimer was still exposed and able to interact with the receptors, akin to native relaxin. This observation is consistent with previous work where prorelaxin with its C-chain intact was still able to interact and signal through the RXFP1 receptor [36, 37]. Interestingly and positively, at RXFP2, the receptor for the related peptide insulin-like peptide 3, the dimer was significantly less active than H2 relaxin itself (Figure 5).

Importantly, in the* in vitro* serum stability assay, the H2 (PEG)_7_ H2 dimer had an improved increase in its half-life compared to the native H2 relaxin (Figures 6(a) and 6(b)), which is probably due to increased molecular shielding. However, as evidenced by the disparity between the* in vitro* serum half-life (ca. 2 h; this study) and reported* in vivo* half-life (a few minutes [14, 15]) of native H2 relaxin, it is clear that renal clearance is a greater issue. It remains to be determined if the increased hydrodynamic volume due to the larger molecular weight of the H2 relaxin dimer will also result in a moderation of its renal clearance. Past experience suggests that this will be the case [33]. Indeed, it was also recently shown that a covalent dimer of exendin-4 had 2.7 times greater increased biological half-life over the native monomer [38].

In conclusion, efficient solid phase peptide synthesis in combination with click chemistry techniques was used to successfully prepare a structurally well-defined homodimer of H2 relaxin, H2 (PEG)_7_ H2. This peptide was shown to possess increased* in vitro* serum stability compared to native H2 relaxin whilst retaining relaxin-like binding affinity, activity, and structural integrity* in vitro*. The dimer peptide also has the potential to slow urinary glomerular filtration rate [30] which will be investigated in the future. Similar approaches can be adopted for the preparation of further increased multimeric forms of H2 relaxin using, for example, a benzene core bearing three or more azido moieties [39] or short PEG-linked dendrons [31].
